# Supplementary material for: The causal relationship between the human gut microbiota and pyogenic arthritis: a Mendelian randomization study
Source: Front Cell Infect Microbiol. 2024 Nov 26;14:1452480. doi: 10.3389/fcimb.2024.1452480 (PMC11629706; doi:10.3389/fcimb.2024.1452480)
Supplement: Supplementary file 1 [file DataSheet1.zip › mendelian test/ebi-a-GCST90027535.csv_forest.pdf]

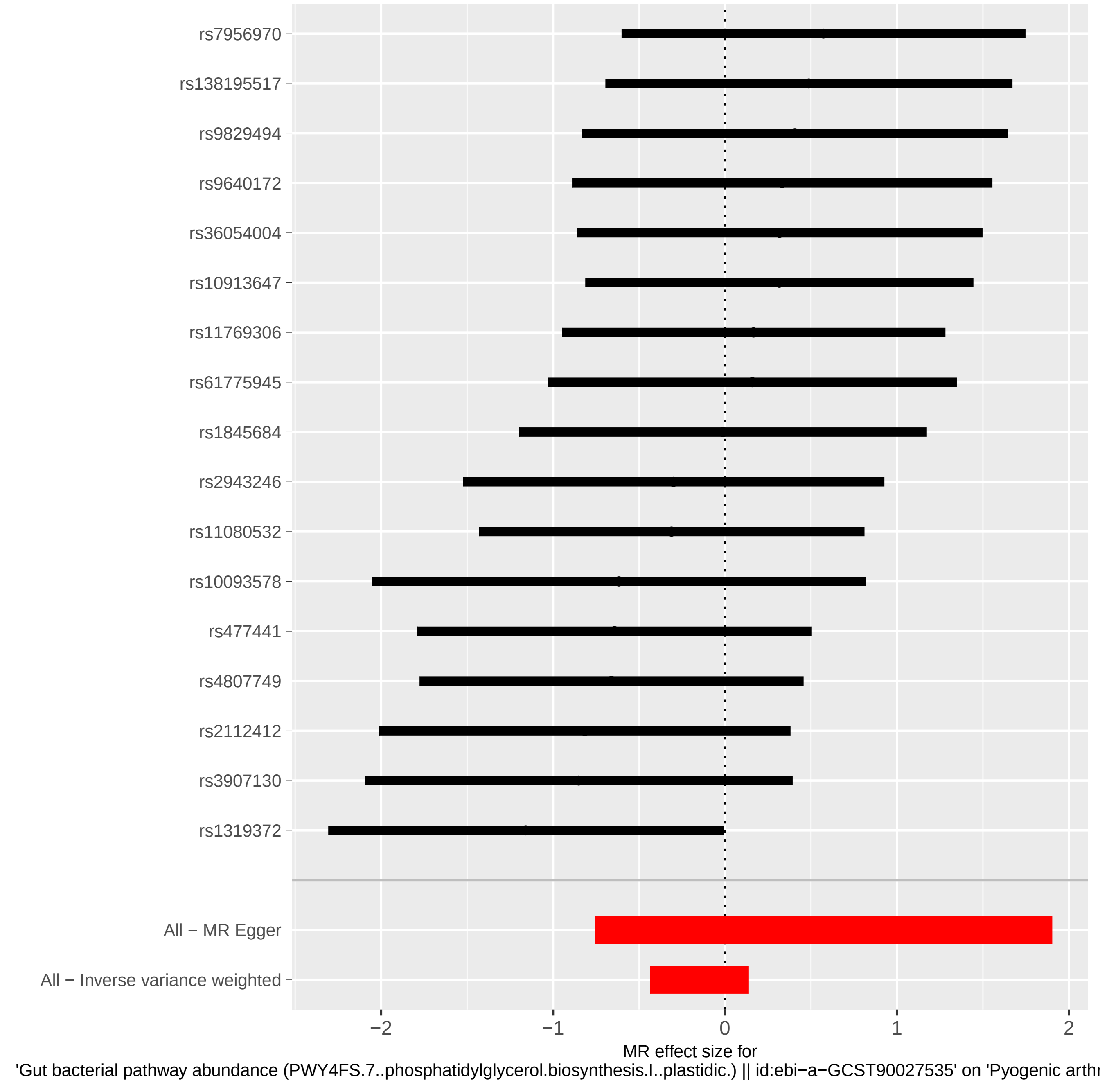

rs7956970
rs138195517
rs9829494
rs9640172
rs36054004
rs10913647
rs11769306
rs61775945
rs1845684
rs2943246
rs11080532
rs10093578
rs477441
rs4807749
rs2112412
rs3907130
rs1319372
All − MR Egger
All − Inverse variance weighted
−2
−1
0
1
2
MR effect size for
'Gut bacterial pathway abundance (PWY4FS.7..phosphatidylglycerol.biosynthesis.I..plastidic.) || id:ebi−a−GCST90027535' on 'Pyogenic arthr
